# Exploring wanting and liking during positive human-animal interactions

**DOI:** 10.1007/s00213-026-07015-w

**Published:** 2026-01-23

**Authors:** Suzanne Truong, Oceane Schmitt, Femke Buisman-Pijlman, Maciej Oczak, Jean-Loup Rault

**Affiliations:** 1 Animal Welfare Science Unit, University of Veterinary Medicine Vienna, Veterinaerplatz 1, 1210 Vienna, Austria; 2 Institute of Animal Breeding and Genetics, Faculty of Agricultural Sciences, Nutritional Sciences, and Environmental Management, University of Giessen, Giessen, Germany; 3 Faculty of Medicine, Dentistry and Health Sciences, University of Melbourne, Victoria, Australia; 4 Discipline of Pharmacology, University of Adelaide, Adelaide, Australia; 5 Precision Livestock Farming Hub, University of Veterinary Medicine Vienna, Vienna, Austria

**Keywords:** Reward system, Amisulpride, Naloxone, Dopamine, Opioid, Receptor antagonist, Social reward, Sus scrofa

## Abstract

**Rationale:**

Dopamine and opioids modulate the “wanting” and “liking” components of reward processes, yet their specific roles in social and human-animal interactions (HAIs) remain unclear.

**Objectives:**

In this pilot study, we aimed to disentangle the roles of dopamine and opioids during HAIs, contrasting them with food reward and novel object test scenarios. We investigated these neurotransmitter systems as proximate mechanisms for “wanting” and “liking” behaviours in domesticated pigs. Additionally, there is a significant knowledge gap on the optimal dosage for the dopamine D2/D3 receptor antagonist amisulpride and mu-opioid receptor antagonist naloxone.

**Methods:**

We administered amisulpride orally and naloxone intranasally at three dosage levels to 30 female pigs using a within-subject design. Each treatment (drug × dose level) was administered prior to 10-min interaction sessions with a familiar human.

**Results:**

Both the high doses of amisulpride (800 mg) and naloxone (80 mg/ml) significantly increased pigs’ latency to first contact with the human compared to pre-test, suggesting both systems modulate the motivation (“wanting”) to interact. However, neither influenced the time pigs spent in contact with the human, contrary to our prediction that blocking opioids would decrease this behaviour as an indicator of the pleasurability (“liking”) of the interaction.

**Conclusion:**

These altered reward-related behaviours along with the absence of side-effects are the first indicators for the safe and effective use of amisulpride and naloxone to non-invasively study reward mechanisms in domesticated animals.

**Supplementary Information:**

The online version contains supplementary material available at https://doi.org/10.1007/s00213-026-07015-w.

## Introduction

Positive human-animal interactions (HAIs) can be sources of rewarding experiences eliciting positive emotions (Rault et al. 2020). Despite the well-known health benefits for both human (Friedmann 2025) and non-human animal (hereafter ‘animal’) (Hemsworth 2003), the neurophysiological basis of positive HAIs remains poorly understood.

Studies have often teased apart the reward process into two distinct components: “wanting” *i.e.* the motivation for the reward during the appetitive phase, and “liking” *i.e.* the hedonic pleasure experienced during the consummatory phase (reviewed in (Pool et al. 2016)), which are involved in the proximate control of behaviour (Gygax 2017). However, “wanting” and “liking” are not completely independent from each other as motivation can be driven by expected pleasantness based on past “liking” experiences (Pool et al. 2016).

Two main neurotransmitters have been implicated as key players in the reward system: dopamine for “wanting”, and opioids for the hedonic “liking” (Barbano & Cador 2007). Pharmacological manipulation is commonly employed to investigate the functions of the dopaminergic and opioidergic systems. For example, studies in rats have demonstrated that mu-opioid agonism in the nucleus accumbens (NAc) shell increases “wanting” of food rewards but increases “liking” when localised in the rostro-dorsal quarter of the medial shell of the NAc (known as the “hedonic hotspot”) (Peciña & Berridge 2005; Castro & Berridge 2014). On the other hand, manipulation of dopamine has been shown to mediate “wanting” but not “liking” of food rewards (Wyvell & Berridge 2000; Robinson et al. 2005). Similarly in humans, oral administration of the dopamine D2/D3 receptor antagonist amisulpride reduced “wanting” *i.e.* motivation and reward anticipation but did not reduce “liking” *i.e.* positive facial reactions during reward consumption, whereas the mu-opioid antagonist naltrexone reduced both (Korb et al. 2020). The opioidergic system has been further implicated in social processes where mu-opioid receptor antagonists reduced social play in juvenile rats (Vanderschuren et al. 1995) and the comforting effect (indicated by number of distress vocalisations) provided by mirrors in socially isolated chicks (Panksepp et al. 1980).

In this study, we investigated the neurophysiological basis of positive human-animal interactions using domesticated pigs given the important implications of HAIs for pig welfare and husbandry (Tallet & Brajon 2024), and the similarities between pig and human anatomy and physiology give our findings high translational potential to inform human studies (Perleberg et al. 2018). Pigs are highly social and able to form close relationships with humans (Graves 1984), often showing voluntary engagement in positive interactions (Rault et al. 2019; Lürzel et al. 2020; Truong et al. 2024). Furthermore, recent evidence from fMRI suggests pigs perceive stroking by a human as pleasurable (Schmitt et al. 2025), altogether highlighting their suitability as a model species.

We hypothesised that pharmacologically blocking dopamine D2/D3 receptors with oral amisulpride would reduce pigs’ motivation to interact (reduced “wanting”, as indicated by increased latency to first contact with the human) and blocking mu-opioid receptors with intranasal naloxone would render the interaction less pleasurable to the pig (reduced “liking”, as indicated by a decrease in the total duration of contact with the human) compared to a pre-test measure.

Given the evidence that dopamine and opioid antagonism affect “wanting” and “liking” of food rewards and novelty-seeking, we used a food reward test and novel object test as “backbones” to further explore the specificity of the effects of the antagonists. We expected a decrease in total duration of foraging (*i.e.* manipulating the treat ball containing the food reward) with mu-opioid receptor blockade (reduced “liking”), and a decrease in total duration of manipulating the novel object as well as increased latency to first contact with the novel object with dopamine D2/D3 receptor blockade compared to pre-test (reduced “wanting”).

Due to the lack of dose–response studies, it is still unclear at what dose amisulpride acts pre-synaptically (agonist effect) or post-synaptically (antagonist effect) and cross-species applications (Webber et al. 2021). Likewise, a dose–response reward study has not yet been conducted with intranasal naloxone. To address this knowledge gap, we tested low, medium, and high doses of both drugs to identify optimal dosages that induce relevant behavioural changes without side effects for future studies. By elucidating the neurobiological mechanisms underlying HAIs and establishing optimal dosages for receptor antagonism in pigs, this study not only advances our understanding of animal behaviour and welfare but also provides a valuable foundation for translational research in human-animal bond studies and broader neuropharmacological investigations.

## Material and methods

### Animals

Thirty prepubertal female pigs (*Sus Scrofa domesticus*; Swiss Large White × Pietrain breed) were enrolled in this study, conducted at the research farm of the University of Veterinary Medicine Vienna (VetFarm ‘Medau’) over two batches (batch 1: 20 test pigs, batch 2: 10 test pigs with an additional 10 pigs to maintain the same group size across both batches). All pigs were recruited at weaning (*i.e.* approximately 28 days of age) as pairs of siblings from different litters, and selected based on health condition, avoiding large weight variations. Enrichment was provided in the form of sawdust and straw, two orange dog toy balls (Airflow ball, Dog Crest, 7.6 cm diameter), and two braided jute ropes (1 m long) hung from the pen fixtures.

### Habituation

Over the course of three weeks, all pigs underwent gradual habituation to human presence, human contact, and to the test pen. The process involved moving the test pig together with two pen mates from their home pen (‘companion pigs’), and upon arriving at the test pen the companion pigs were moved into the companion pen adjacent to the test pen and the test pig into the test pen. The companion pen and test pen were separated by a partition with a ‘social window’ (50 × 25 cm) allowing visual, auditory and olfactory access to minimise potential distress due to social separation for the test pig (Kroell et al. 2025). The general interaction procedure during the habituation sessions was as follows: the interacting human, already seated in the corner opposite to the pen door, gently encouraged the pig to approach upon it entering the test pen using a soft talking voice and small hand gestures. If the pig approached and initiated contact, she slowly presented her hand to the pig’s snout, then gently applied tactile contact (stroking or scratching) starting on the head area, then moving to the neck and towards the rear. This also included belly-rubbing if the pig rolled over and exposed its belly (Rault et al. 2019). After the interaction sessions, the pigs remained in the test pen without the human for an additional 5 min to habituate to being alone (with companion pigs in the adjacent pen), which was the length of time of the additional food reward and novel object tests that were to be conducted during the testing period. On the last two days of habituation, pigs were introduced to the treat ball (Lesfit, Yiwu Baoda Garment Accessories Co., Ltd, Yiwu City, China) containing food rewards (*i.e.* banana chips) during the 5 min following the interaction sessions to avoid the effect of novelty.

### Training

In the second week of habituation, pigs underwent daily training in their home pen by another experimenter (O.S.) to receive 0.2 ml of 0.9% saline intranasally with a half dose in each nostril using a mucosal atomiser device (Teleflex MAD Nasal, Teleflex Medical GmbH, Fellbach, Germany) connected to a 1 ml syringe according to a procedure used previously for oxytocin intranasal administration in pigs (Rault et al. 2013). The pigs were trained to sit with their head slightly up and accept the insertion of the syringe in both nostrils without manual restraint. Only positive reinforcement was used with food reward (*i.e.* apple pieces).

During the last three days of habituation, the pigs were trained to accept liquid (*i.e.* water) from a 10 ml syringe to train for oral drug administration. The experimenter allowed the pigs to approach and voluntarily place their mouth on the syringe, then delivered the content to the pigs. On the last day of habituation, apple juice was given instead of water to reduce novelty effects as this was the palatable liquid food to be mixed with amisulpride.

### Drug and dose selection

Amisulpride (AMI) is an orally-available, highly selective dopamine D2/D3 receptor antagonist that has been used to study social rewards in humans (Korb et al. 2020; Massaccesi et al. 2024) and in pharmacokinetic studies in rodents (Perrault et al. 1997; Schoemaker et al. 1997). Naloxone (NAL) is a fast-acting opioid antagonist with a high affinity for mu-opioid receptors (Krieter et al. 2016), rapidly occupying brain mu-opioid receptors when administered intranasally (monkey: Saccone et al. 2016; human: Johansson et al. 2019; dog: Wahler et al. 2019). The low (L), medium (M), and high (H) doses (AMI-L: 200 mg, AMI-M: 500 mg, AMI-H: 800 mg, NAL-L: 2 mg, NAL-M: 4 mg, NAL-H: 8 mg) were selected based on previous studies having used them in humans and non-human animals via the same routes of administration used in this study (Table S1). These doses were either within the recommended range approved for clinical use in humans and/or had not shown side effects in healthy individuals in previous studies. The optimal dose was considered based on efficacy (*i.e.* pigs expressing relevant behavioural changes) and safety (*i.e.* no behaviours indicating side effects). The drug biperiden (Akineton® 2 mg) (Desma GmbH, Mainz-Kastel, Germany) was kept on-hand to quickly reverse any side effects of amisulpride (see Table 1 for abnormal behaviours).

**Table 1:** Definitions of measures obtained from behavioural observations and computer vision techniques

Test	Measure	Definition
*Human-animal interaction test*	Latency to first contact with human	Time from start of test (when the pig’s shoulders pass the wooden beam at the test pen door) till first point of physical contact between the pig and human. This contact could be initiated by either the pig or the human on any part of the body
	Total duration of contact with human	Duration of pig and human having any part of their bodies in physical contact with each other during the session
	Variation in speed	Variance of the speed of movement of the pig during the session
*Food reward test*	Latency to first contact with treat ball^1^	Time from start of test (when the pig’s shoulders pass the wooden beam at the test pen door) till first point of contact between pig snout and treat ball
	Proportion of time foraging	Proportion of time of the session where the pig is manipulating the treat ball with its snout, mouth, or has its head down where the treat ball is presumed to be in contact underneath the head (out of view), or the pig is presumed to be eating the banana chips that have fallen out of the treat ball near the treat ball
*Novel object test*	Latency to first contact with novel object^1^	Time from start of test (when the pig’s shoulders pass the wooden beam at the test pen door) till first point of contact between pig snout and novel object
	Proportion of time manipulating the novel object	Proportion of time of the session where the pig is manipulating the novel object with its snout, mouth or has its head down where the object is presumed to be in contact (e.g. underneath the head)
	Total duration of looking at novel object	Duration of the proxy of the pig’s bifocal line of vision intersecting with the bounding box indicating location of novel object during the session
*Abnormal behaviours*	Non-forward locomotion^2,3^	Non-locomotory leg movements, backward locomotion and rotation around hind legs (> 180°)
	Posturing^2,3^	Standing in an awkward, odd or un-physiological position > 2 s
	Head/facial/oral activity^2,3^	Head shaking, jerking head movements, facial dyskinesias and chewing that is not associated with exploration, licking or yawning
	Distant look^2,3^	Standing without focusing and with a distant look > 2 s
	Stereotypic behaviour^2,3^	Any behaviour that is repetitive and with little variation and no obvious function). This includes signs of restlessness such as pacing
	Tremor^3^	Rhythmic and involuntary movement (shaking) of any body part
	Salivation^3^	Saliva discharge from the snout
	Vomiting^3^	Retching and expulsion of the stomach contents

^1^These variables were excluded from statistical analyses due to a floor effect. See Sect. "Statistical analyses" for more details

^2^ Lind et al. (2005)

^3^These variables were excluded from statistical analyses due to rare occurrences

### Drug preparation

Amisulpride was administered orally, but in different forms in the batches due to observed aversion (e.g. expelling the solution from the mouth and subsequently avoiding the oral syringe). For Batch 1, SOLIAN® liquid oral solution (Sanofi-Aventis, Quetigny, France) mixed with apple juice or Nestlé® condensed milk was prepared fresh on testing days. For Batch 2, Amisu® tablets (G.L. Pharma GmbH, Lannach, Austria) were crushed according to the dosages and stored in 10 ml centrifuge tubes. The crushed powder was mixed with apple juice and Nestlé® condensed milk to 10 ml volume on testing days (see Table S2a for detailed mixing ratios). This aversion-based adjustment ensured consistent drug administration across both batches while addressing the pigs' preferences.

The naloxone solutions were prepared on the pre-test day. Naloxone hydrochloride dihydrate (Siegfried PharmaChemikalien Minden GmbH, Minden, Germany) was weighed using a precision scale (Satorius Analytic AC121S, Satorius AG, Göttingen, Germany), transferred to 1.5 ml Eppendorf tubes, and mixed up with a 0.9% saline solution according to the formulations described in Table S2b. The solutions were mixed with a vortex mixer (Vortex Genie 2 mode G-560E, Scientific Industries, New York, USA), immediately placed on ice and stored at 4 °C (see Table S2b for full protocol). The intranasal syringes were prepared on the morning of each testing day, each containing half-doses (0.1 ml) to be delivered in each nostril.

### Drug administration

**Fig. 1:**
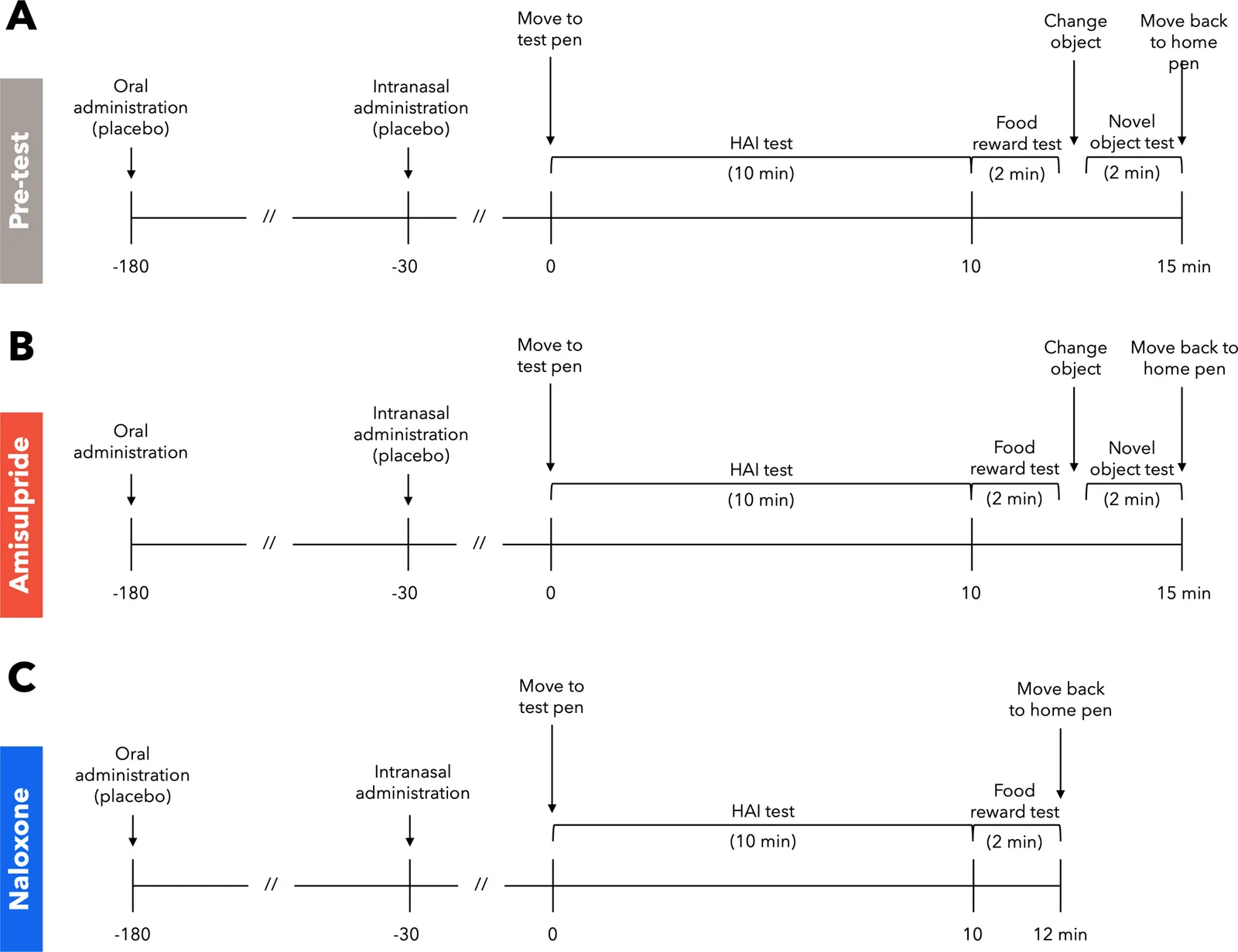
Drug administration and test timeline according to the treatment: (**A**) pre-test (placebo only), (**B**) dopamine receptor antagonist amisulpride, (**C**) opioid receptor antagonist naloxone. The placebo for oral administration was apple juice and the placebo for intranasal administration was saline. Administration was timed with peak concentration expected to be reached during the human-animal interaction (HAI) tests: 3 h prior to testing for amisulpride, 30 min for naloxone. The 10-min HAI test was followed by a 2-min food reward test then a 2-min novel object test. The novel object tests were conducted only for the pre-test and amisulpride treatments to validate previous findings on the effect of dopamine antagonism on response to novelty from a similar test conducted in pigs (Lind et al. 2005)

Drug administration took place in the home pen prior to testing. Administration was timed to match the time at which the drug was expected to reach peak concentration during the HAI tests (Fig. 1). Therefore, amisulpride was administered orally 3 h (Korb et al. 2020) before testing (Fig. 1B), and naloxone was administered intranasally 30 min (McDonald et al. 2018; Krieter et al. 2019) before testing (Fig. 1C). To control for the effects of handling between oral and intranasal administration, pigs received a placebo at each time point of administration for each drug they were not being tested for that day. The placebo for oral administration was apple juice, and the placebo for intranasal administration was saline.

### Testing

The testing period spanned across three weeks. Testing was conducted once every three days to allow for a washout period of two days between tests based on the elimination half-life of each drug (naloxone: Wahler et al. 2019; amisulpride: Sparshatt et al. 2009) to avoid potential carryover effects. A within-subject, counterbalanced design was used where every pig was subject to all drug × dose combinations (total of six tests per pig). All pigs started with pre-test (P) on the first day where they were administered only placebos (Fig. 1A), and alternated drug on subsequent test days with half starting with naloxone and the other half starting with amisulpride. The order of dose levels was pseudo-randomised resulting in a different test order for every pig.

#### Human-animal interaction tests

For the HAI tests, the interacting human entered the test pen and sat in the corner opposite to the pen door. The test pig and two companion pigs were then moved as done during habituation. The human (blinded to the treatments) remained seated and interacted with the pig as she had done during habituation, gently encouraging the pig to approach and provided gentle tactile and verbal contact for 10 min.

#### Food reward test

After the HAI test, the test pig was guided out of the test pen and the interacting human placed the treat ball containing banana chips as food rewards in a set position. The interacting human then exited the pen, and the test pig was once again guided back into the test pen to begin the food reward test. The food reward test lasted two minutes, where the pig was allowed to freely interact with the treat ball containing food rewards.

#### Novel object test

Following the food reward test, the test pig was guided out of the test pen by the handler, after which the interacting human removed the treat ball and any leftover banana chips, and replaced it with one of four novel objects (1: red KONG Wobbler™ (KONG Company, Colorado, USA), 2: blue and red KONG Gyro (KONG Company, Colorado, USA), 3: yellow ring (FIT + FUN, MultiFit Tiernahrungs GmbH, Krefeld, Germany), 4: pink bone (TEDi Warenhandels GmbH, Vienna, Austria); Figure S1). The order of novel objects was pseudo-randomised to be balanced across tests and different for each pig, so that each object was novel for each test.

### Behavioural analyses

The test sessions were video recorded using cameras connected to a server (Hikvision DS-2CD5046GO-AP; Hangzhou Hikvision Electronics Co., Ltd., Hangzhou, China), fixed on the ceiling above the test pen, which allowed for post-hoc behavioural analysis and extraction of behavioural measures using computer vision techniques. Behaviours were analysed using continuous focal sampling method (Bateson & Martin 2021) (BORIS software v.8.20.4, see Table 1) by observers who were blinded to the treatment conditions. Inter- and intra-observer agreement were substantial to near-perfect (Table S3). Although the manipulation of the treat ball and consumption of banana chips are presumed to be two separate mechanisms (appetitive (“wanting”) and consummatory (“liking”) respectively), we combined these two behaviours into one (‘foraging’) due to the inability to differentiate them from the video footage.

#### Variables extracted using computer vision

The ‘variation in speed’ during the HAI tests and ‘total duration of looking at novel object’ during the novel object tests were calculated using computer vision techniques, using the skeleton-based image feature extraction method described in Oczak et al. (2024). The procedure was composed of: 1) Camera calibration, which allowed estimation of size of pixels in the images in the units of the metric system of measurement *i.e.* centimetres; 2) Detection of a pig and novel object with the YOLOv8 object detection model; 3) Detection of pig’s key points with the VitPose key point detection model; 4) Calculation of Euclidean distance between centroids of bounding boxes with detected pig with sampling rate of 1 fps, which allowed estimation of speed of movement of pigs in cm/s; 5) Calculation of variance in speed of movement in cm^2^/s^2^; 6) Calculation of proxy of pig’s bifocal line of vision based on key points neck and snout; 7) Calculation of duration of intersection of pig’s bifocal line of vision with bounding box indicating location of novel object.

### Statistical analyses

Statistical analyses were performed using the R statistical software (version 4.4.1) (R Core Team 2024). Each behavioural variable was analysed using separate (generalised) linear mixed models ((G)LMMs). We used a full model comprised of dose as a fixed effect (levels: P, L, M, H) and subject as a random effect. We also included batch as a fixed effect and day as a covariate and random slope within subject to control for potential confounding effects. For the novel object test variables, the object type was also included as a fixed effect. Given this was a pilot study designed to explore the dose–response relationships for amisulpride and naloxone, we did not perform adjustments for multiple comparisons. We acknowledge the increased risk of Type I error, and that any *p* values reported should be interpreted as exploratory and suggestive of associations. An effect was deemed significant if the *p* value was below 0.05. The latency data from the food reward test and novel object test were heavily right-skewed due to a floor effect (probably due to the small size of the test pen) with most pigs making contact within 2 s (92.4% food reward test sessions, 63.5% novel object test sessions). Therefore, any differences would not be considered biologically relevant, and we deemed these data not suitable for statistical analyses (see summary in Table S4). All results are presented as estimated mean ± standard error. A complete description of the statistical analyses can be found in the Supplementary Material.

## Results

### Abnormal behaviours

One pig showed one occurrence of vomiting during one HAI test session. No other abnormal behaviours indicating side effects were observed.

### Human-animal interaction test

**Fig. 2:**
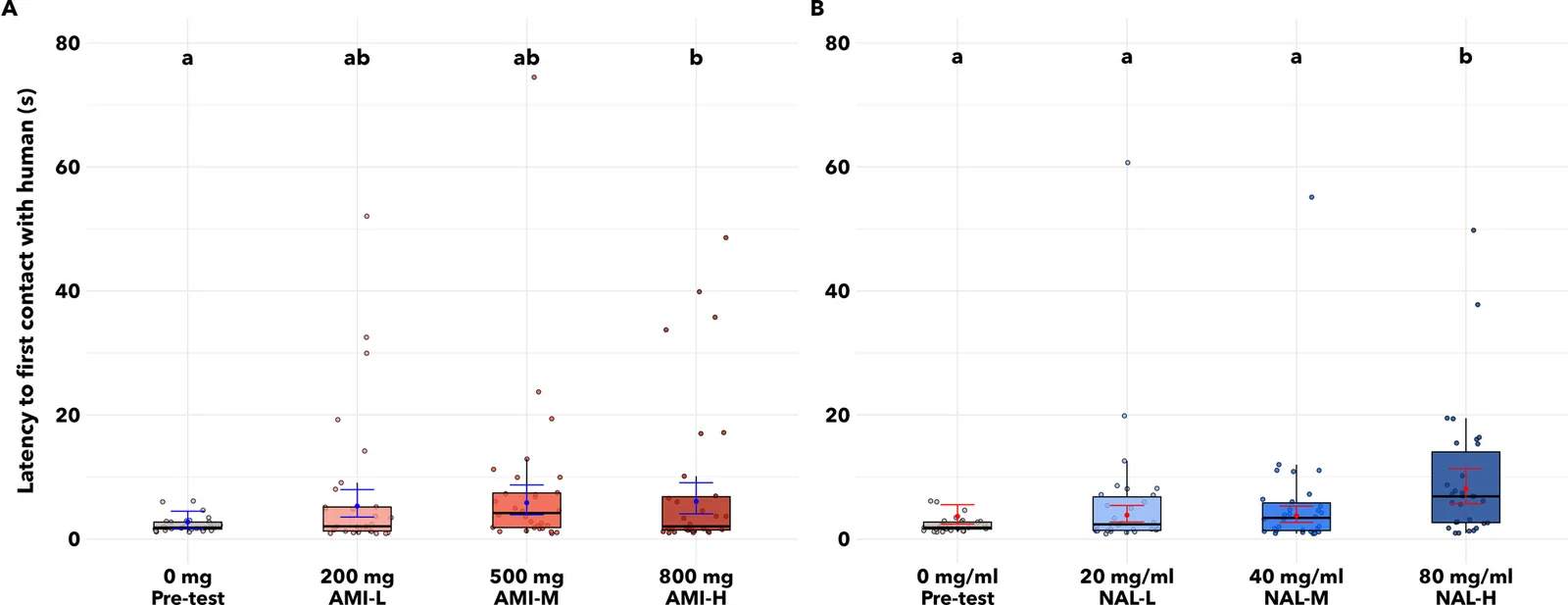
Post-hoc pairwise comparisons of latency to first contact with human (*n* = 30) during the human-animal interaction test sessions after administration of the dopamine receptor antagonist amisulpride (**A**) or the opioid receptor antagonist naloxone (**B**). Blue/red dot and error bar = estimated mean and associated 95% confidence interval; horizontal black line within box = raw median; jitter dots = raw data points; lower and upper edge of box = 1^st^ and 3rd quartile; whiskers = data within 1.5*interquartile range (IQR). Grey = pre-test; AMI = amisulpride; NAL = naloxone; L = low; M = medium; H = high. Different letters (a, b) indicate significant differences (*p* < 0.05) between treatments (pre-test, L, M, H) within a given drug

There was a significant dose effect on pigs’ latency to first point of physical contact with the human for both drugs (amisulpride: χ^2^(3) = 7.864, *p* = 0.049; naloxone: χ^2^(3) = 18.461, *p* < 0.001; Fig. 2). Pigs given the high dose (800 mg) of amisulpride showed significantly higher latency to first contact with the human compared to pre-test (Fig. 2A and Table S5a). With naloxone, pigs given the high dose (80 mg/ml) showed significantly higher latency to first contact with the human compared to pre-test, the low dose (20 mg/ml), and the medium dose (40 mg/ml) (Fig. 2B). There was a significant positive relationship between latency and day for naloxone (χ2(1) = 7.469, *p* = 0.006), but not for amisulpride (*p* = 0.543).

Pigs did not significantly differ in their total duration in contact with the human according to dose or day for either drug (Table S5b).

There was no significant dose effect of amisulpride on pigs’ variation in speed (F_1,95_ = 2.772, *p* = 0.099; Table S5c).

### Food reward test

There was no significant dose effect of naloxone or amisulpride on the proportion of time spent foraging, nor an effect of day for either drug (Figure S2, Table S6).

### Novel object test

**Fig. 3:**
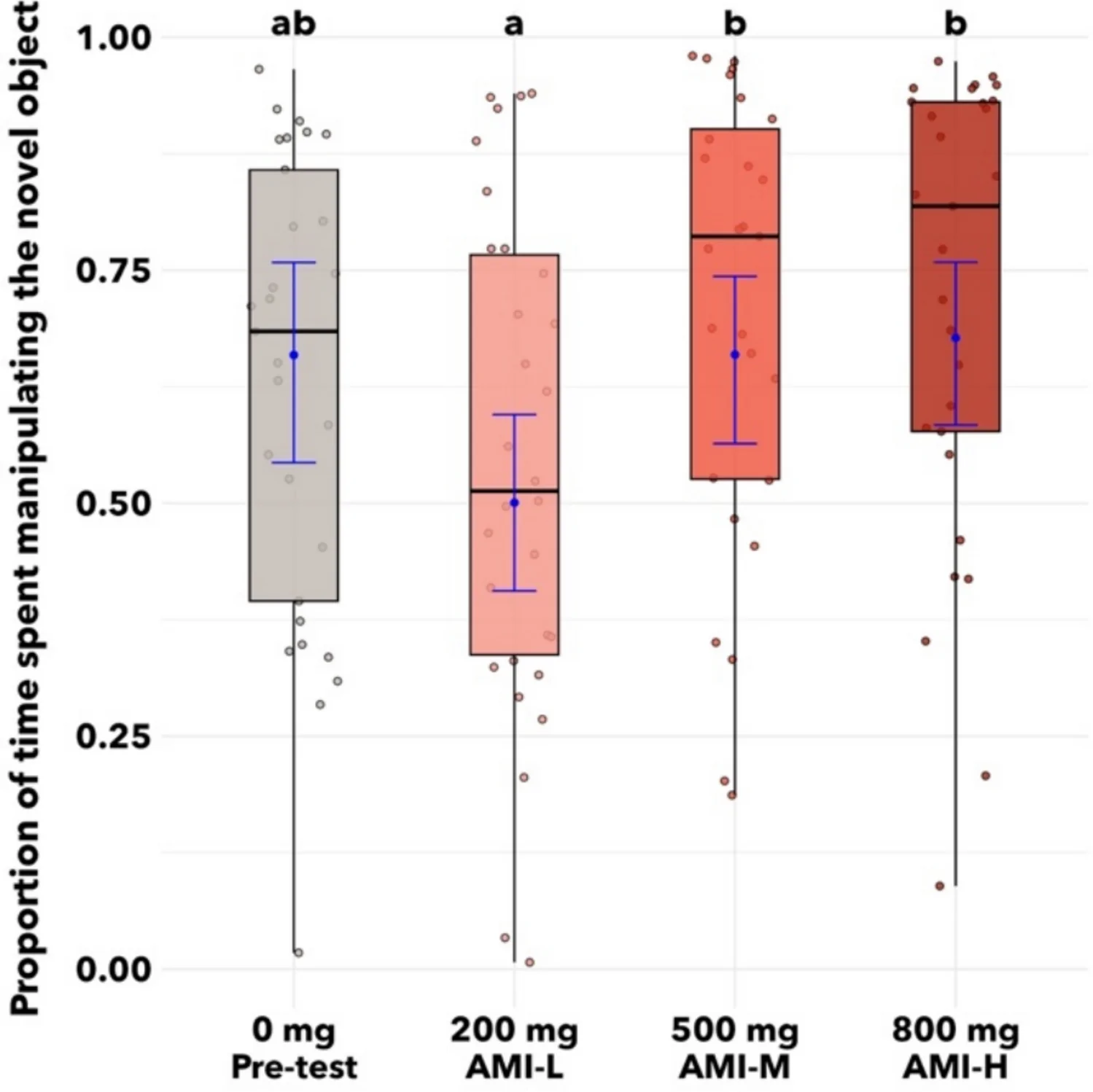
Post-hoc pairwise comparisons of the proportion of time spent manipulating the novel object (*n* = 30) during the novel object test sessions after administration of the dopamine receptor antagonist amisulpride. Blue dot and error bar = estimated mean and associated 95% confidence interval; horizontal black line within box = raw median; jitter dots = raw data points; lower and upper edge of box = 1^st^ and 3^rd^ quartile; whiskers = data within 1.5*interquartile range (IQR). AMI = amisulpride; L = low; M = medium; H = high. Different letters (a, b) indicate significant differences (*p* < 0.05) between treatments (pre-test, L, M, H) within a given drug

There was a significant dose effect of amisulpride (χ^2^(3) = 9.99, *p* = 0.019; Fig. 3) on the proportion of time pigs spent manipulating the novel object, with lower proportions when given the low dose (200 mg) compared to the medium (500 mg) and high (800 mg) doses. There was no significant effect of day (Table S7a).

There were no significant effects of dose or day on the total duration of looking at the novel object (Table S7b). There was a significant effect of object type (F_3,80_ = 6.05, *p* = 0.001), where pigs spent more time looking at the KONG Gyro compared to the red KONG Wobbler™ and the pink bone. They also spent more time looking at the yellow ring compared to the pink bone.

## Discussion

This pilot study investigated the dose–response relationship of the dopamine D2/D3 receptor antagonist amisulpride and mu-opioid receptor antagonist naloxone in relation to reward-related behaviour in pigs. In line with the literature, the high doses of both amisulpride and naloxone significantly increased pigs’ latency to first contact with the human (*i.e.* decreased “wanting”) compared to pre-test. In addition, latencies increased across the testing days for naloxone, suggesting a cumulative effect that could be due to pharmacological carryover or influence from previous experience. Contrary to predictions, the total duration of contact with the human (“liking”) was not significantly different between doses for either drug.

The increased latency to first contact with the human following administration of the high dose of both drugs suggests that both dopamine D2 and mu-opioid receptors are involved in modulating motivation (“wanting”). Although we did not have predictions regarding the effect of naloxone on latency, this is consistent with previous literature that opioids are also involved in “wanting” mechanisms (Peciña, 2008). Importantly, this also suggests that latency as a behavioural measure of motivation is unspecific in relation to components of the reward system and cannot be used alone to differentiate the effects of the dopaminergic or opioidergic system on “wanting”. To control for the potential side-effect of dopamine antagonism on locomotor function, we measured the variation in speed and found no significant differences according to doses.

The effect of ‘day’ on latency to first contact with the human for naloxone suggests there is a potential cumulative effect, which could be due to either pharmacological carryover (*i.e.* drug did not completely clear by the next test) or the effect of previous experience (*i.e.* decreased “liking” from previous interaction reduced motivation in subsequent interactions) as has been observed before in human-pig interactions (Truong et al. 2024). As all test orders were pseudo-randomised resulting in every pig having its own testing order, a potential order effect (e.g. pigs showing a stronger response depending on which dose they started with) could not be disentangled from the day effect. In addition to “wanting” and “liking”, there is a third reward component that has been described in the literature: “learning”, predictions about future rewards based on previous experiences (Berridge & Kringelbach 2008). It is possible that, due to the effect of receptor antagonism by naloxone on motivation in previous sessions, this could have reduced the reward value of the interactions and decreased their motivation to interact in subsequent sessions, rendering the interactions less desirable over time.

Importantly, the lack of difference in total duration of contact with the human suggests that this behavioural measure was either not influenced by the dopaminergic or opioidergic system in our study, or not sensitive enough to manifest in an observable difference. It could also be that no behavioural differences were observed despite underlying neurobiological differences (Massaccesi et al. 2024), although this could not be confirmed in our study. Alternatively, the oxytocinergic system could have been involved as opioid antagonism enhances oxytocin release, and the two systems together strongly influence social behaviour (reviewed in (Putnam & Chang 2022)). It could be interpreted that an observed effect on our measure of “wanting” along with the lack of difference in our measure of “liking” suggests these mechanisms are dissociable (Soutschek et al. 2021), however, without the observable effect of naloxone on our measure of “liking” it is difficult to discern this. Variability could have overshadowed potential differences, considering the moderate coefficient of variation values for this variable (Table S8).

Although not significant, naloxone showed a tendency to reduce the proportion of time spent foraging during the food reward tests whereas amisulpride did not. Here, there is evidence to support that the mechanisms are dissociable, in line with a previous study that showed naltrexone (another non-selective opioid receptor antagonist) but not amisulpride affected the “liking” of food rewards (Korb et al. 2020). It is unclear why only the medium dose but not the high dose of naloxone showed this effect. A potential explanation could be that at higher doses, there are greater chances of kappa-opioid receptor blockade (Trøstheim et al. 2023). This could have counteracted the effects of mu-opioid blockade as kappa-opioid antagonism can stimulate dopamine release (reviewed in (Bruijnzeel 2009)). However, we did not observe this same pattern with the latency to make first contact with the human, potentially ruling out this explanation.

Our results from the novel object test differed from a previous study that investigated the effect of dopamine antagonism on novelty using haloperidol in pigs (Lind et al. 2005). We found higher duration of contact with the novel object under the medium and high doses compared to the low dose (but not pre-test), whereas Lind et al. (2005) found haloperidol decreased contact time. We also did not find differences with the duration of looking at the novel object due to amisulpride, only due to object type, whereas Lind et al. (2005) found haloperidol increased the duration of looking at the novel object. This could be due to the unintended consequence of the social window allowing premature visual exposure to the objects. As dopamine D2 antagonists generally increase unconditioned fear (Alves et al. 2024), it is likely that it was this unintended visual exposure that resulted in decreased fear responses to novelty and therefore less inhibition to interacting with the novel objects. Although the low dose of amisulpride significantly reduced the proportion of time pigs spent manipulating the novel object, this appeared to be confounded by the type of object due to a failure to balance the object presentation order across doses (Table S9). The effect of object type on both the duration of manipulating the object and looking at the object suggests that the pigs perceived differences in the objects’ affordances and perceptual features (Heyser & Chemero 2012). Although this perceptual difference was to be expected, its confounding effect is perhaps made more pronounced as it obscures the effect of amisulpride.

There are limitations in this study that warrant attention. Firstly, the unanticipated aversiveness of amisulpride resulted in moderate leakage (*i.e.* expulsion of amisulpride solution from the mouth) in some pigs (Table S10). This could have biased our results towards a weaker response as some pigs did not receive the full dose of the drugs. Future studies should improve the palatability of the drug to ensure successful delivery of the full dosages to allow proper assessment of effects on behaviour and potential side effects. As we only observed one occurrence of vomiting and no other abnormal behaviours, it is difficult to ascertain whether this was caused by the drug. Secondly, the camera setup did not allow us to differentiate “wanting” and “liking” behaviours during the food reward test. Future studies should consider a different camera setup that enables measuring palatability and orofacial expression (Figueroa et al. 2019) and facial affect coding (Camerlink et al. 2018) to have more direct and specific indices of hedonic affect. In addition, employing a study design that allows for a direct comparison between social and non-social rewards, such as that of Korb et al. (2020), would better enable elucidating the specificity of the drugs. Finally, the within-subject, multiple dose design employed to minimise animal numbers and account for individual variation potentially introduced a cumulative effect as seen with the ‘day’ effect for naloxone. As a pilot study, it was necessary to conduct multiple doses within one individual as the individual differences in pharmacokinetics could be large (Li et al. 2020; Saari et al. 2024). In future, studies could utilise a single dose (based on our findings) to avoid this cumulative effect, and subsequent analysis using rank order test or Bayesian statistics to deal with individual variation. This should ideally also include both sexes to explore potential sex differences, as previous studies have found sex differences in reaction to novelty (Lind et al. 2005) and oxytocin administration relating to social behaviour (reviewed in Rault et al. (2017)) that could interplay with the effects of dopamine and opioids.

This study provides preliminary evidence of altered pig behaviour towards rewarding situations by dopamine and opioid receptor antagonism. Both amisulpride and naloxone reduced pigs’ motivation (“wanting”) to interact with the human, but neither reduced the “liking” of the interaction. Our findings can help to inform future studies in determining the safe and effective dosages of oral amisulpride and intranasal naloxone for the study of reward mechanisms.

## Supplementary Information

Below is the link to the electronic supplementary material.

Supplementary file1 (DOCX 461 KB)

## Data Availability

The data pertaining to the results presented in this study are available at: https://phaidra.vetmeduni.ac.at/o:4099.
