# Supplementary material for: Exploring wanting and liking during positive human-animal interactions
Source: Psychopharmacology (Berl). 2026 Jan 23;243(11):2577–87. doi: 10.1007/s00213-026-07015-w (PMC13710614; doi:10.1007/s00213-026-07015-w)
Supplement: Supplementary file 1 [file 213_2026_7015_MOESM1_ESM.docx]

**Supplementary Material**

Exploring wanting and liking during positive human-animal interactions

Suzanne Truong^1^, Oceane Schmitt^1,2^, Femke Buisman-Pijlman^3,4^, Maciej Oczak^5^, Jean-Loup Rault^1,*^

^1^ Animal Welfare Science Unit, University of Veterinary Medicine Vienna, Vienna, Austria

^2^ Institute of Animal Breeding and Genetics, Faculty of Agricultural Sciences, Nutritional Sciences, and Environmental Management, University of Giessen, Giessen, Germany

^3^ Faculty of Medicine, Dentistry and Health Sciences, University of Melbourne, Victoria, Australia

^4^ Discipline of Pharmacology, University of Adelaide, Adelaide, Australia

^5^ Precision Livestock Farming Hub, University of Veterinary Medicine Vienna, Vienna, Austria

*Correspondence to: Veterinaerplatz 1, A-1210 Vienna, Austria

*Email address:* Jean-Loup.Rault@vetmeduni.ac.at (J.-L. Rault)

**Detailed Methodology**

**Animals, housing and feeding.** The pigs were housed in groups of 10, with one sibling of each litter pair in each pen (2.45 × 3.82 m) composed of grey plastic walls, partly-slatted concrete flooring (slatted area = 2.43 × 1.18 m; 30% of pen area) equipped with a multi-place feeder (eight places), one drinker, and a shelter area (2.43 × 1.00 m) with a roof 0.94 m above the ground. The pigs were fed a standard weaner diet (17.5% crude protein, 7% crude fat; Garant-Tiernahrung GmbH, Austria) topped with supplement (Biotronic Top3 and ProbioBac; Biomin®, Austria) *ad libitium* throughout the experimental period. Spot cleaning of the pen (*i.e.* removal of faeces and soiled straw) and topping up of straw was done daily. Room temperature was automatically regulated by a temperature control system (CB2012, Strijbos Climatech, de Wit AgroHandel GmbH; Austria) and thermal comfort was ensured by floor heating.

**Habituation.** Eight researchers were involved in conducting this study: one interacting human (S.T.: 159 cm, black overalls), one trainer (O.S.: 173 cm, blue overalls), and three handlers per batch (Batch 1: J.W., F.D., G.F.; Batch 2: T.Z., M.N., C.B.; all wearing red overalls). Aside from the first group habituation in the home pen, only the interacting human was involved in the interaction sessions throughout habituation and testing. The handlers moved the pigs to and from their home pen, gently guiding movement using a plastic livestock board. Habituation involved a stepwise process, starting with group habituation in their home pen for the first day, then habituation in the test pen progressed from groups of 10 pigs in the test pen, to groups of 5 (plus 5 companion pigs in adjacent pen), to pairs (plus 2 companion pigs in adjacent pen), to being alone (plus 2 companion pigs in adjacent pen).

**Statistical analyses.** The data were subsetted per drug as they were intended to target different receptors and were not expected to be directly comparable. Distribution of data was inspected prior to fitting the models to ascertain distribution was approximately normal. The variables ‘total duration of contact with the human’, ‘looking at the novel object’, and ‘variation in speed’ (log-transformed) were fitted with LMMs based on Gaussian distribution using the ‘lme4’ package (version 1.1.35.5) (Bates et al., 2015). The latency variables were heavily right-skewed and fitted with GLMM based on gamma distributions with a log link function using the ‘glmmTMB’ package (version 1.1.9) (Brooks et al., 2017) as the data were not censored. The duration of foraging and duration of manipulating the novel object were analysed as proportions because these data were heavily left-skewed and fitted with GLMM based on beta distribution with a logit link function using the ‘glmmTMB’ package (version 1.1.9) (Brooks et al., 2017). Model assumptions were checked by visual inspection of the QQ-plot (Field, 2005) of residuals and residuals plotted against fitted values (Quinn & Keough, 2002) for the LMMs. The ‘DHARMa’ package (version 0.4.6) (Hartig, 2016) was used to inspect the QQ plot residuals for quantile deviations and test for dispersion for the GLMMs. Multicollinearity was checked using the ‘performance’ package (version 0.12.3) (Lüdecke et al., 2021) and did not appear to be an issue (maximum Variance Inflation Factor: 2.23) (Quinn & Keough, 2002). Model stability was assessed using a function provided by Roger Mundry. All the GLMMs showed good model stability, but the LMMs showed some model convergence warnings and caution should be taken when interpreting these results. The ‘emmeans’ package (version 1.10.4) (Lenth, 2023) was used to obtain the estimated marginal means, standard errors, and confidence limits for the levels of each factor and conduct posthoc pairwise comparisons with Tukey adjustment. Significance for each effect was determined by a Type III ANOVA F-test with Satterthwaite approximation (Luke, 2017) for the LMMs, and chi-square test comparing Akaike Information Criterion (AIC) of models with each predictor (dose, batch, day) removed using the drop1 function (Chambers & Hastie, 1992) for the GLMMs. For the novel object test data, 5 sessions were excluded: for the following reasons: 2 due to the pigs accidentally being given the same object twice, 1 due to a pig accessing an object outside of testing, 1 due to failed video recording of the session, and 1 was considered an outlier being the only session out of 120 sessions where the pig did not make contact with the novel object at all.

**References**

Bates, D., Mächler, M., Bolker, B., & Walker, S. (2015). Fitting linear mixed-effects models using lme4. *Journal of Statistical Software*, *67*(1), 1–48. https://doi.org/10.18637/jss.v067.i01

Brooks, M. E., Kristensen, K., van Benthem, K. J., Magnusson, A., Berg, C. W., Nielsen, A., Skaug, H. J., Maechler, M., & Bolker, B. M. (2017). glmmTMB balances speed and flexibility among packages for zero-inflated generalized linear mixed modeling. *The R Journal*, *9*(2), 378–400. https://doi.org/10.32614/RJ-2017-066

Chambers, J., & Hastie, T. (1992). Linear models. Chapter 4 of statistical models in S. *Wadsworth & Brooks/Cole*, *1992*, 96–138.

Field, A. (2005). Repeated-measures designs. *Discovering Statistics Using SPSS*, 427–482.

Hartig, F. (2016). DHARMa: Residual diagnostics for hierarchical (multi-level/mixed) regression models. *CRAN: Contributed Packages*.

Lenth, R. V. (2023). *emmeans: Estimated marginal means, aka least-squares means* [Manual]. https://CRAN.R-project.org/package=emmeans

Lüdecke, D., Ben-Shachar, M., Patil, I., Waggoner, P., & Makowski, D. (2021). performance: An R Package for Assessment, Comparison and Testing of Statistical Models. *Journal of Open Source Software*, *6*(60), 3139. https://doi.org/10.21105/joss.03139

Luke, S. G. (2017). Evaluating significance in linear mixed-effects models in R. *Behavior Research Methods*, *49*(4), 1494–1502. https://doi.org/10.3758/s13428-016-0809-y

Quinn, G. P., & Keough, M. J. (2002). *Experimental Design and Data Analysis for Biologists*. Cambridge University Press.

**Table S1.** References for dose selection.

| Treatment | Drug | Dose level | Dose | Route of administration | References |
| --- | --- | --- | --- | --- | --- |
| NAL-L | Naloxone | Low | 2 mg (20mg/ml) | Intranasal | 1, 2, 4 mg (humans)^1^;  2, 4 mg (humans)^2^; 2, 4, 8 mg (humans)^3^; 4 mg (dogs)^4^ |
| NAL-M | Naloxone | Med | 4 mg (40mg/ml) | Intranasal |  |
| NAL-H | Naloxone | High | 8 mg (80mg/ml) | Intranasal |  |
| AMI-L | Amisulpride | Low | 200 mg | Oral | Recommended range: 200 mg (human children)^5^  400-800mg (humans)^6–8^;  400 mg (humans)^9-10^;  400, 800, 1200mg (humans)^11^; 800mg (humans)^11,12^ |
| AMI-M | Amisulpride | Med | 500 mg* | Oral |  |
| AMI-H | Amisulpride | High | 800 mg | Oral |  |
| *The 500 mg dose of amisulpride was recommended because the commonly used 400 mg dose is at the border of pre-/post-synaptic effects (G. Silani, pers. comm., 15 September 2022). | | | | | |
| **References** 1. McDonald, R. et al. Pharmacokinetics of concentrated naloxone nasal spray for opioid overdose reversal: Phase I healthy volunteer study. Addiction 113, 484–493 (2018). 2. Johansson, J. et al. Intranasal naloxone rapidly occupies brain mu-opioid receptors in human subjects. Neuropsychopharmacology 44, 1667–1673 (2019). 3. Krieter, P. et al. Pharmacokinetic Properties and Human Use Characteristics of an FDA-Approved Intranasal Naloxone Product for the Treatment of Opioid Overdose. J. Clin. Pharmacol. 1243–1253 (2016) doi:10.1002/jcph.759. 4. Wahler, B. M. et al. Pharmacokinetics and pharmacodynamics of intranasal and intravenous naloxone hydrochloride administration in healthy dogs. Am. J. Vet. Res. 80, 696–701 (2019). 5. Chung, Y. S. et al. Comparative cardiometabolic risk of antipsychotics in children, adolescents and young adults. Eur. Child Adolesc. Psychiatry 30, 769–783 (2021). 6. Li, L., Li, L., Shang, D. W., Wen, Y. G. & Ning, Y. P. A systematic review and combined meta-analysis of concentration of oral amisulpride. British Journal of Clinical Pharmacology vol. 86 668–678 Preprint at https://doi.org/10.1111/bcp.14246 (2020). 7. Huang, S. et al. Modeling and simulation for individualized therapy of amisulpride in chinese patients with schizophrenia: Focus on interindividual variability, therapeutic reference range and the laboratory alert level. Drug Des. Devel. Ther. 15, 3903–3913 (2021). 8. Sparshatt, A., Taylor, D., Patel, M. X. & Kapur, S. Amisulpride - Dose, plasma concentration, occupancy and response: Implications for therapeutic drug monitoring. Acta Psychiatrica Scandinavica vol. 120 416–428 Preprint at https://doi.org/10.1111/j.1600-0447.2009.01429.x (2009). 9. Weber, S. C. et al. Dopamine D2/3- and μ-opioid receptor antagonists reduce cue-induced responding and reward impulsivity in humans. Transl. Psychiatry 6, (2016). 10. Korb, S. et al. Dopaminergic and opioidergic regulation during anticipation and consumption of social and nonsocial rewards. eLife 9, 1–22 (2020). 11. Peuskens, J. et al. Amisulpride vs. risperidone in the treatment of acute exacerbations of schizophrenia. Psychiatry Res. 88, 107–117 (1999). 12. Möller, H. J., Boyer, P., Fleurot, O. & Rein, W. Improvement of acute exacerbations of schizophrenia with amisulpride: a comparison with haloperidol. Psychopharmacology (Berl.) 132, 396–401 (1997). | | | | | |

**Table S2a.** Drug preparation protocol.

**Amisulpride** (SOLIAN®) 100 mg/ml liquid solution

| Dosage | Total volume to be delivered | Formulation | Volume of amisulpride | Volume of apple juice |
| --- | --- | --- | --- | --- |
| 0 mg | 10 ml | 100 mg/ml | 0 ml | 10 ml |
| 200 mg | 10 ml | 100 mg/ml | 2 ml | 8 ml |
| 500 mg | 10 ml | 100 mg/ml | 5 ml | 5 ml |
| 800 mg | 10 ml | 100 mg/ml | 8 ml | 2 ml |

Prepare: 10 ml syringe, amisulpride oral solution (SOLIAN®), apple juice (or Nestle® condensed milk)

Testing day

1. Using the 10 ml syringe, draw the volume of apple juice specified for the particular dosage treatment (*e.g.* 2 ml apple juice for 800 mg dosage).
2. With the same syringe already containing apple juice, draw the volume of amisulpride specified for the particular dosage treatment (*e.g.* 8 ml amisulpride for 800 mg dosage).

**Amisulpride** (Amisu®) 200 mg and 400 mg tablets

Prepare: Amisulpride tablets (Amisu®), 10 ml centrifuge tubes, pestle and mortar, permament marker, 10 ml syringe, apple juice, Nestle® condensed milk, stirrer, cup

1. Crush the amisulpride tablets according to the dosages (200 mg, 500 mg, 800 mg) using the pestle and mortar. Prepare only one dosage at a time.
2. Transfer the amisulpride powder to a centrifuge tube. Each centrifuge tube contains only the exact amount for one dose.
3. Label the tube with the dosage using the permanent marker.
4. Store at room temperature until testing day.

Testing day

5. Mix the powder and 10 ml of apple juice and Nestle® condensed milk in a cup with a stirrer.
6. Draw the amisulpride solution using the 10 ml oral syringe.

**Table S2b.** Drug preparation protocol.

**Naloxone**

| Dosage | Formulation | Amount of Naloxone HCl per full dose | Total amount of Naloxone HCl required (for 20 pigs) | Total amount of Naloxone HCl for double* | Total volume of 0.9% Saline for double |
| --- | --- | --- | --- | --- | --- |
| 0 mg | 0 mg/ml | 0 x 0.2 = 0 mg | 0 mg x 20 = 0 mg | 0 mg | 8 ml |
| 2 mg | 10 mg/ml | 10 x 0.2 = 2 mg | 2 mg x 20 = 40 mg | 80 mg | 8 ml |
| 4 mg | 20 mg/ml | 20 x 0.2 = 4 mg | 4 mg x 20 = 80 mg | 160 mg | 8 ml |
| 8 mg | 40 mg/ml | 40 x 0.2 = 8 mg | 8 mg x 20 = 160 mg | 320 mg | 8 ml |

* Double the total amount needed was prepared as back-up in case of a missed administration.

Prepare: Naloxone hydrochloride dihydrate, 0.9% saline solution, spatula, weighing paper, precision scale, 3x 10 ml centrifuge tubes, 30x 1.5 ml Eppendorf tubes, permanent marker, vortex machine, Styrofoam box with ice

1. Place the weighing paper on the precision scale and tare the scale.
2. Using the spatula, transfer a small amount of Naloxone Hydrochloride Dihydrate to the weighing paper and weigh the appropriate amount required for the specific formulation (*e.g.* 80 mg for the 10 mg/ml formulation).
3. Transfer the weighed amount of Naloxone Hydrochloride Dihydrate to the centrifuge tube.
4. Add the specified volume of saline solution to the centrifuge tube.
5. Stir with the spatula. Allow solution to sit for 1 min.
6. Pipette 0.8 ml aliquots of each Naloxone formulation into the Eppendorf tubes.
7. Use the vortex machine to mix the Eppendorf tubes at 400 rpm for 10 sec before placing in ice.
8. Store the Styrofoam box containing all tubes in the refrigerator at 4°C.

Testing day

9. Draw 0.1 ml of the naloxone solution from the pre-prepared Eppendorf tube containing the correct dosage. Prepare this three times (one syringe for each nostril + one back-up).

**Table S3.** Inter- and intra-observer reliability: Cohen’s Kappa (κ) values.

| Test | Inter-observer κ | Intra-observer κ |
| --- | --- | --- |
| Human-animal interaction | 0.903 | 0.986 |
| Food reward | 0.740 | 0.909 |
| Novel object | 0.879 | 0.962 |

**Figure S1.** Novel objects used during the novel object tests. 1: red KONG Wobbler™, 2: blue and red KONG Gyro, 3: yellow ring, 4: pink bone.

**Table S4.** Descriptive statistics on latencies during the food reward test and novel object tests.

| **Latency to first physical contact with food reward** | | | | |  |  |  |
| --- | --- | --- | --- | --- | --- | --- | --- |
| Drug | Term | Level | <2 s | ≥ 2 s & < 10 s |  |  |  |
| Amisulpride |  |  |  |  |  |  |  |
|  | Dose |  |  |  |  |  |  |
|  |  | P | 21 | 9 |  |  |  |
|  |  | L | 29 | 1 |  |  |  |
|  |  | M | 28 | 2 |  |  |  |
|  |  | H | 29 | 1 |  |  |  |
|  | Batch |  |  |  |  |  |  |
|  |  | B1 | 73 | 7 |  |  |  |
|  |  | B2 | 34 | 6 |  |  |  |
| Naloxone |  |  |  |  |  |  |  |
|  | Dose |  |  |  |  |  |  |
|  |  | P | 21 | 9 |  |  |  |
|  |  | L | 29 | 1 |  |  |  |
|  |  | M | 29 | 1 |  |  |  |
|  |  | H | 29 | 1 |  |  |  |
|  | Batch |  |  |  |  |  |  |
|  |  | B1 | 73 | 7 |  |  |  |
|  |  | B2 | 35 | 5 |  |  |  |
| **Latency to first physical contact with novel object** | | | | | |  |  |
| Drug | Term | Level | < 2 s | ≥ 2 s & < 10 s | ≥ 10 s |  |  |
| Amisulpride |  |  |  |  |  |  |  |
|  | Dose |  |  |  |  |  |  |
|  |  | P | 12 | 16 | 1 |  |  |
|  |  | L | 20 | 9 | 1 |  |  |
|  |  | M | 23 | 4 | 0 |  |  |
|  |  | H | 18 | 11 | 0 |  |  |
|  | Batch |  |  |  |  |  |  |
|  |  | B1 | 48 | 27 | 1 |  |  |
|  |  | B2 | 25 | 13 | 1 |  |  |
|  | Object type |  |  |  |  |  |  |
|  |  | 1 | 20 | 9 | 1 |  |  |
|  |  | 2 | 21 | 8 | 0 |  |  |
|  |  | 3 | 16 | 14 | 0 |  |  |
|  |  | 4 | 16 | 9 | 1 |  |  |

P = pre-test; L = low; M = medium; H = high; B1 = batch 1; B2 = batch 2; Object type: 1 = red KONG Wobbler™, 2 = blue and red KONG Gyro, 3 = yellow ring, 4 = pink bone. The latency data were categorised as follows: ‘<2 s’ = pig approached and made contact with the food reward/novel object immediately upon entering the test pen; ‘≥ 2 s & < 10 s’ = pig approached the food reward/object quickly but not immediately upon entering the test pen; ‘≥ 10 s’ = pig did not approach the food reward/object quickly.

**Table S5a.** Model results for pigs’ behaviours during the human-animal interaction tests.

| **Latency to first physical contact with human** | | | | | | | | | |
| --- | --- | --- | --- | --- | --- | --- | --- | --- | --- |
| Drug | Term | Level | Response (s) | SE | Df | Asymp LCL | Asymp UCL | χ² | Pr(>Chi) |
| Amisulpride |  |  |  |  |  |  |  |  |  |
|  | Dose |  |  |  |  |  |  | 7.864 | 0.049 |
|  |  | P | 2.76 | 0.68 | Inf | 1.70 | 4.48 |  |  |
|  |  | L | 5.31 | 1.10 | Inf | 3.53 | 7.97 |  |  |
|  |  | M | 5.84 | 1.20 | Inf | 3.91 | 8.73 |  |  |
|  |  | H | 6.07 | 1.25 | Inf | 4.06 | 9.08 |  |  |
|  | Batch |  |  |  |  |  |  | 2.224 | 0.136 |
|  |  | B1 | 3.91 | 0.63 | Inf | 2.85 | 5.37 |  |  |
|  |  | B2 | 5.83 | 1.27 | Inf | 3.80 | 8.94 |  |  |
|  | Day |  |  |  |  |  |  | 0.370 | 0.543 |
| Naloxone |  |  |  |  |  |  |  |  |  |
|  | Dose |  |  |  |  |  |  | 18.461 | <.001 |
|  |  | B | 3.66 | 0.77 | Inf | 2.42 | 5.53 |  |  |
|  |  | L | 3.85 | 0.67 | Inf | 2.74 | 5.41 |  |  |
|  |  | M | 3.77 | 0.66 | Inf | 2.68 | 5.30 |  |  |
|  |  | H | 8.04 | 1.41 | Inf | 5.70 | 11.35 |  |  |
|  | Batch |  |  |  |  |  |  | 1.794 | 0.180 |
|  |  | B1 | 3.94 | 0.51 | Inf | 3.05 | 5.07 |  |  |
|  |  | B2 | 5.25 | 0.93 | Inf | 3.71 | 7.43 |  |  |
|  | Day |  |  |  |  |  |  | 7.469 | 0.006 |
| P = pre-test; L = low; M = medium; H = high; B1 = batch 1; B2 = batch 2; SE = standard error; Df = degrees of freedom; Asymp = asymptotic; LCL = lower confidence limit; UCL = upper confidence limit. Response = estimated response, calculated from post-hoc pairwise comparisons with Tukey adjustment for dose (P/L, P/M, P/H, L/M, L/H, M/H), and batch (B1/B2). The chi-square (χ²) value and probability of chi-square statistic (Pr(>Chi)) calculated from chi-square tests comparing Akaike Information Criterion (AIC) of the models with each predictor (dose, batch, day) removed from the GLMM. Day as an integer was included as a covariate in the model. | | | | | | | | | |

**Table S5b.** Model results for pigs’ behaviours during the human-animal interaction tests.

| **Total duration of physical contact with human** | | | | | | | | | |
| --- | --- | --- | --- | --- | --- | --- | --- | --- | --- |
| Drug | Term | Level | Emmean (s) | SE | Df | LCL | UCL | F value | Pr(>F) |
| Amisulpride |  |  |  |  |  |  |  |  |  |
|  | Dose |  |  |  |  |  |  | 1.475 | 0.229 |
|  |  | P | 381 | 21.8 | 84 | 338 | 425 |  |  |
|  |  | L | 368 | 18.7 | 79 | 331 | 406 |  |  |
|  |  | M | 358 | 18.6 | 80 | 321 | 395 |  |  |
|  |  | H | 334 | 18.7 | 80 | 297 | 371 |  |  |
|  | Batch |  |  |  |  |  |  | 0.004 | 0.950 |
|  |  | B1 | 360 | 15.2 | 28 | 329 | 391 |  |  |
|  |  | B2 | 361 | 21.4 | 28 | 318 | 405 |  |  |
|  | Day |  |  |  |  |  |  | 0.520 | 0.474 |
| Naloxone |  |  |  |  |  |  |  |  |  |
|  | Dose |  |  |  |  |  |  | 0.720 | 0.543 |
|  |  | P | 375 | 21.9 | 103 | 332 | 419 |  |  |
|  |  | L | 341 | 18.5 | 85 | 304 | 377 |  |  |
|  |  | M | 345 | 18.3 | 83 | 309 | 382 |  |  |
|  |  | H | 339 | 18.4 | 84 | 302 | 375 |  |  |
|  | Batch |  |  |  |  |  |  | 0.064 | 0.802 |
|  |  | B1 | 347 | 15 | 26 | 316 | 378 |  |  |
|  |  | B2 | 353 | 21.2 | 26 | 310 | 397 |  |  |
|  | Day |  |  |  |  |  |  | 0.074 | 0.786 |
| P = pre-test; L = low; M = medium; H = high; B1 = batch 1; B2 = batch 2; SE = standard error; Df = degrees of freedom; LCL = lower confidence limit; UCL = upper confidence limit. Emmean = estimated mean, calculated from post-hoc pairwise comparisons with Tukey adjustment for dose (P/L, P/M, P/H, L/M, L/H, M/H), and batch (B1/B2). F-value and P-value (Pr(>F)) calculated from Type III Analysis of Variance with Satterthwaite's method for the overall effect of each term in the GLMM. Day as an integer was included as a covariate in the model. | | | | | | | | | |

**Table S5c.** Model results for pigs’ behaviours during the human-animal interaction tests.

| **Variation in speed** | | | | | | | | | |
| --- | --- | --- | --- | --- | --- | --- | --- | --- | --- |
| Drug | Term | Level | Emmean (m/s) | SE | Df | LCL | UCL | F value | Pr(>F) |
| Amisulpride |  |  |  |  |  |  |  |  |  |
|  | Dose |  |  |  |  |  |  | 2.086 | 0.108 |
|  |  | P | 1.12E-05 | 9.15E-07 | 106 | 9.41E-06 | 1.30E-05 |  |  |
|  |  | L | 1.01E-05 | 6.55E-07 | 87 | 8.80E-06 | 1.14E-05 |  |  |
|  |  | M | 8.94E-06 | 5.87E-07 | 87 | 7.77E-06 | 1.01E-05 |  |  |
|  |  | H | 8.90E-06 | 5.91E-07 | 87 | 7.73E-06 | 1.01E-05 |  |  |
|  | Batch |  |  |  |  |  |  | 4.920 | 0.035 |
|  |  | B1 | 9.08E-06 | 3.49E-07 | 110 | 8.39E-06 | 9.77E-06 |  |  |
|  |  | B2 | 1.05E-05 | 5.59E-07 | 87 | 9.39E-06 | 1.16E-05 |  |  |
|  | Day |  |  |  |  |  |  | 2.772 | 0.099 |
| Naloxone |  |  |  |  |  |  |  |  |  |
|  | Dose |  |  |  |  |  |  | 0.875 | 0.457 |
|  |  | P | 1.04E-05 | 8.39E-07 | 86 | 8.70E-06 | 1.20E-05 |  |  |
|  |  | L | 9.79E-06 | 6.50E-07 | 63 | 8.49E-06 | 1.11E-05 |  |  |
|  |  | M | 9.20E-06 | 6.09E-07 | 62 | 7.98E-06 | 1.04E-05 |  |  |
|  |  | H | 8.98E-06 | 5.98E-07 | 63 | 7.78E-06 | 1.02E-05 |  |  |
|  | Batch |  |  |  |  |  |  | 2.093 | 0.159 |
|  |  | B1 | 9.01E-06 | 4.31E-07 | 90 | 8.16E-06 | 9.87E-06 |  |  |
|  |  | B2 | 1.02E-05 | 6.78E-07 | 62 | 8.80E-06 | 1.15E-05 |  |  |
|  | Day |  |  |  |  |  |  | 0.078 | 0.780 |
| P = pre-test; L = low; M = medium; H = high; B1 = batch 1; B2 = batch 2; SE = standard error; Df = degrees of freedom; LCL = lower confidence limit; UCL = upper confidence limit. Emmean = estimated mean, calculated from post-hoc pairwise comparisons with Tukey adjustment for dose (P/L, P/M, P/H, L/M, L/H, M/H), and batch (B1/B2). F-value and P-value (Pr(>F)) calculated from Type III Analysis of Variance with Satterthwaite's method for the overall effect of each term in the GLMM. Day as an integer was included as a covariate in the model. | | | | | | | | | |

**Figure S2.** Post-hoc pairwise comparisons of the proportion of time spent foraging (n = 30) during the food reward test sessions after administration of the dopamine receptor antagonist amisulpride (A) or the opioid receptor antagonist naloxone (B). Red dot and error bar = estimated mean and associated 95% confidence interval; horizontal black line within box = raw median; jitter dots = raw data points; lower and upper edge of box = 1st and 3rd quartile; whiskers = data within 1.5*interquartile range (IQR). NAL = naloxone; L = low; M = medium; H = high. Statistical significance between treatments (pre-test, L, M, H) within a given drug indicated by the following symbols: †0.05> p ≤ 0.1,*p < .05; **p < .01; ***p < .001.

**Table S6.** Model results for pigs’ behaviours during the food reward tests.

| **Proportion of time spent foraging** | | | | | | | | | |
| --- | --- | --- | --- | --- | --- | --- | --- | --- | --- |
| Drug | Term | Level | Response | SE | Df | Asymp LCL | Asymp UCL | χ² | Pr(>Chi) |
| Amisulpride |  |  |  |  |  |  |  |  |  |
|  | Dose |  |  |  |  |  |  | 0.984 | 0.805 |
|  |  | P | 0.85 | 0.036 | Inf | 0.77 | 0.91 |  |  |
|  |  | L | 0.81 | 0.035 | Inf | 0.73 | 0.87 |  |  |
|  |  | M | 0.83 | 0.033 | Inf | 0.76 | 0.89 |  |  |
|  |  | H | 0.84 | 0.032 | Inf | 0.77 | 0.89 |  |  |
|  | Batch |  |  |  |  |  |  | 0.576 | 0.448 |
|  |  | B1 | 0.82 | 0.028 | Inf | 0.76 | 0.87 |  |  |
|  |  | B2 | 0.85 | 0.034 | Inf | 0.77 | 0.91 |  |  |
|  | Day |  |  |  |  |  |  | 3.515 | 0.061 |
| Naloxone |  |  |  |  |  |  |  |  |  |
|  | Dose |  |  |  |  |  |  | 6.966 | 0.073 |
|  |  | P | 0.87 | 0.031 | Inf | 0.80 | 0.92 |  |  |
|  |  | L | 0.83 | 0.032 | Inf | 0.75 | 0.88 |  |  |
|  |  | M | 0.76 | 0.039 | Inf | 0.68 | 0.83 |  |  |
|  |  | H | 0.79 | 0.037 | Inf | 0.70 | 0.85 |  |  |
|  | Batch |  |  |  |  |  |  | 0.038 | 0.846 |
|  |  | B1 | 0.81 | 0.029 | Inf | 0.75 | 0.86 |  |  |
|  |  | B2 | 0.82 | 0.038 | Inf | 0.73 | 0.88 |  |  |
|  | Day |  |  |  |  |  |  | 1.539 | 0.215 |
| P = pre-test; L = low; M = medium; H = high; B1 = batch 1; B2 = batch 2; SE = standard error; Df = degrees of freedom; Asymp = asymptotic; LCL = lower confidence limit; UCL = upper confidence limit. Response = estimated response, calculated from post-hoc pairwise comparisons with Tukey adjustment for dose (P/L, P/M, P/H, L/M, L/H, M/H), and batch (B1/B2). The chi-square (χ²) value and probability of chi-square statistic (Pr(>Chi)) calculated from chi-square tests comparing Akaike Information Criterion (AIC) of the models with each predictor (dose, batch, day) removed from the GLMM. Day as an integer was included as a covariate in the model. | | | | | | | | | |

**Table S7a.** Model results for pigs’ behaviours during the novel object tests.

| **Proportion of time spent manipulating the novel object** | | | | | | | | | |
| --- | --- | --- | --- | --- | --- | --- | --- | --- | --- |
| Drug | Term | Level | Response | SE | Df | Asymp LCL | Asymp UCL | χ² | Pr(>Chi) |
| Amisulpride |  |  |  |  |  |  |  |  |  |
|  | Dose |  |  |  |  |  |  | 9.523 | 0.023 |
|  |  | P | 0.65 | 0.056 | Inf | 0.54 | 0.75 |  |  |
|  |  | L | 0.50 | 0.049 | Inf | 0.41 | 0.60 |  |  |
|  |  | M | 0.66 | 0.046 | Inf | 0.57 | 0.75 |  |  |
|  |  | H | 0.67 | 0.045 | Inf | 0.58 | 0.76 |  |  |
|  | Batch |  |  |  |  |  |  | 4.307 | 0.038 |
|  |  | B1 | 0.68 | 0.031 | Inf | 0.62 | 0.74 |  |  |
|  |  | B2 | 0.57 | 0.046 | Inf | 0.48 | 0.65 |  |  |
|  | Day |  |  |  |  |  |  | 2.669 | 0.102 |
|  | Object type |  |  |  |  |  |  | 7.281 | 0.063 |
|  |  | 1 | 0.56 | 0.048 | Inf | 0.47 | 0.65 |  |  |
|  |  | 2 | 0.60 | 0.046 | Inf | 0.51 | 0.69 |  |  |
|  |  | 3 | 0.71 | 0.040 | Inf | 0.63 | 0.79 |  |  |
|  |  | 4 | 0.62 | 0.046 | Inf | 0.53 | 0.71 |  |  |
| P = pre-test; L = low; M = medium; H = high; B1 = batch 1; B2 = batch 2; Object type: 1 = red KONG Wobbler™, 2 = blue and red KONG Gyro, 3 = yellow ring, 4 = pink bone; SE = standard error; Df = degrees of freedom; Asymp = asymptotic; LCL = lower confidence limit; UCL = upper confidence limit. Response = estimated response, calculated from post-hoc pairwise comparisons with Tukey adjustment for dose (P/L, P/M, P/H, L/M, L/H, M/H), and batch (B1/B2). The chi-square (χ²) value and probability of chi-square statistic (Pr(>Chi)) calculated from chi-square tests comparing Akaike Information Criterion (AIC) of the models with each predictor (dose, batch, day, object type) removed from the GLMM. Day as an integer was included as a covariate in the model. | | | | | | | | | |

**Table S7b.** Model results for pigs’ behaviours during the novel object tests.

| **Total duration of looking at novel object** | | | | | | | | | |
| --- | --- | --- | --- | --- | --- | --- | --- | --- | --- |
| Drug | Term | Level | Emmean (s) | SE | Df | LCL | UCL | F value | Pr(>F) |
| Amisulpride |  |  |  |  |  |  |  |  |  |
|  | Dose |  |  |  |  |  |  | 1.325 | 0.272 |
|  |  | P | 48.2 | 4.29 | 100 | 39.7 | 56.7 |  |  |
|  |  | L | 48.4 | 3.47 | 104 | 41.5 | 55.3 |  |  |
|  |  | M | 56.2 | 3.55 | 104 | 49.1 | 63.2 |  |  |
|  |  | H | 48.8 | 3.48 | 103 | 41.9 | 55.7 |  |  |
|  | Batch |  |  |  |  |  |  | 0.061 | 0.807 |
|  |  | B1 | 49.9 | 2.18 | 28 | 45.4 | 54.4 |  |  |
|  |  | B2 | 50.8 | 3.04 | 26 | 44.6 | 57.1 |  |  |
|  | Day |  |  |  |  |  |  | 0.975 | 0.327 |
|  | Object type |  |  |  |  |  |  | 6.054 | 0.001 |
|  |  | 1 | 45.6 | 3.34 | 104 | 39.0 | 52.3 |  |  |
|  |  | 2 | 58.8 | 3.33 | 104 | 52.2 | 65.5 |  |  |
|  |  | 3 | 55.3 | 3.26 | 103 | 48.8 | 61.7 |  |  |
|  |  | 4 | 41.8 | 3.56 | 105 | 34.7 | 48.8 |  |  |
| P = pre-test; L = low; M = medium; H = high; B1 = batch 1; B2 = batch 2; Object type: 1 = red KONG Wobbler™, 2 = blue and red KONG Gyro, 3 = yellow ring, 4 = pink bone; SE = standard error; Df = degrees of freedom; LCL = lower confidence limit; UCL = upper confidence limit. Emmean = estimated mean, calculated from post-hoc pairwise comparisons with Tukey adjustment for dose (P/L, P/M, P/H, L/M, L/H, M/H), and batch (B1/B2). F-value and P-value (Pr(>F)) calculated from Type III Analysis of Variance with Satterthwaite's method for the overall effect of each term (dose, batch, day, object type) in the GLMM. Day as an integer was included as a covariate in the model. | | | | | | | | | |

**Table S8.** Coefficient of variation (CV) of total duration of contact with human during the human-animal interaction tests. P = pre-test; L = Low; M = Medium; H = High.

| Drug | Dose | CV (%) |
| --- | --- | --- |
| Amisulpride |  |  |
|  | P | 23.2 |
|  | L | 21.8 |
|  | M | 30.5 |
|  | H | 33.8 |
| Naloxone |  |  |
|  | P | 23.2 |
|  | L | 24.5 |
|  | M | 31.8 |
|  | H | 27.8 |

**Table S9.** Number of sessions per object type per dose. P = pre-test; L = Low; M = Medium; H = High; 1 = red KONG Wobbler™; 2 = blue and red KONG Gyro; 3 = yellow ring; 4 = pink bone.

|  | Dose | | | |
| --- | --- | --- | --- | --- |
| Object type | P | L | M | H |
| 1 | 7 | 13 | 6 | 4 |
| 2 | 8 | 6 | 8 | 7 |
| 3 | 8 | 7 | 9 | 6 |
| 4 | 6 | 4 | 4 | 12 |

**Table S10.** Summary of estimated amisulpride leakage: number of pigs per dose level per estimated volume of leakage. The leakage volume (in ml) was approximated by collecting the expelled liquid with a hand under the pig's chin and comparing this volume with a measured volume of water in a syringe. Dose levels: P = pre-test; L = Low; M = Medium; H = High

|  | Dose level | | | |  |
| --- | --- | --- | --- | --- | --- |
| Estimated  leakage (ml) | P | L | M | H | Total number of pigs per leakage volume |
| 0 | 30 | 1 | 0 | 1 | 32 |
| 0.5 | 0 | 3 | 0 | 1 | 4 |
| 1 | 0 | 9 | 3 | 4 | 16 |
| 1.5 | 0 | 2 | 1 | 6 | 9 |
| 2 | 0 | 9 | 12 | 4 | 25 |
| 2.5 | 0 | 2 | 5 | 4 | 11 |
| 3 | 0 | 2 | 5 | 5 | 12 |
| 3.5 | 0 | 0 | 2 | 3 | 5 |
| 4 | 0 | 1 | 0 | 1 | 2 |
| 5 | 0 | 1 | 1 | 1 | 3 |
| 8 | 0 | 0 | 1 | 0 | 1 |
